# Distinguishing the role of positivity bias, cognitive impairment and emotional reactivity in the deontological preference in multiple sclerosis during moral dilemmas: a social cognition study protocol

**DOI:** 10.3389/fpsyg.2024.1404876

**Published:** 2024-07-18

**Authors:** Laurent Zikos, Béatrice Degraeve, Antonio Pinti, Julien Poupart, Laurène Norberciak, Arnaud Kwiatkowski, Cécile Donze, Bruno Lenne

**Affiliations:** ^1^Experience, Transhumanism, Human Interactions, Care & Society (ETHICS - EA7446), Lille Catholic University, Lille, France; ^2^Laboratoire Science de l’Information-Communication (LSC/DeVisu), Université Polytechnique Hauts-de-France, Valenciennes, France; ^3^Neurology Department, Groupement des Hôpitaux de l’Institut Catholique de Lille (GHICL), Lille Catholic University, Lille, France; ^4^Rehabilitation Department, Groupement des Hôpitaux de l’Institut Catholique de Lille (GHICL), Lille Catholic University, Lille, France

**Keywords:** psychology, cognition, cognitive neuroscience, social cognition, moral judgement, multiple sclerosis, neurodegenerative diseases, neuropsychology

## Abstract

**Background:**

Multiple sclerosis (MS) is a chronic inflammatory and neurodegenerative disease of the central nervous system characterized by a broad and unpredictable range of symptoms, including cognitive and sociocognitive dysfunction. Among these social-cognitive functions, moral judgment has been explored in persons with MS (PwMS) using moral dilemmas, where participants must decide whether to sacrifice one person to save a greater number. Opting for such a sacrifice reflects utilitarian reasoning (sacrificing one for the benefit of many is deemed acceptable), while refusing reflects deontological reasoning (such sacrifice is considered morally wrong). Compared to controls, PwMS have been shown to make greater deontological moral choices in such dilemmas.

**Objectives:**

While PwMS have demonstrated a higher tendency for deontological moral choices in moral dilemmas compared to controls, the underlying determinants of this reasoning pattern remain unclear. In this project, we aim to investigate cognitive, emotional, and motivational factors that may explain deontological decision-making in MS.

**Methods and analysis:**

We will recruit a sample of 45 PwMS and 45 controls aged 18–55 years. The type of response, deontological or utilitarian, to a series of 20 vignettes of moral dilemmas will constitute the primary outcomes. Global cognitive performance, positivity bias, alexithymia and empathy levels as well as emotional reactivity measured by electrodermal activity (EDA) during moral dilemmas will be secondary outcomes.

**Ethics and dissemination:**

Ethics approval was granted by a national ethical committee (CPP Ouest III, national number 2023-A00447-38). The project is sponsored by the ARSEP Foundation. Findings will be presented at national and international conferences, as well as published in peer-reviewed scientific journals.

## Background

Multiple sclerosis (MS) is a chronic, inflammatory, autoimmune and demyelinating disease that affects young adults (between 20 and 40 years old) and has a significant impact on patients’ quality of life. The main disorders found in these patients are motor disorders, somesthesia, visual disorders, sexual disorders and chronic fatigue. Among the various symptoms encountered, cognitive disturbances are frequent (40–70% of patients; Kujala, 1997), appear early and have a major impact on the socio-professional life and quality of life of patients (Chiaravalloti and DeLuca, 2008; Grzegorski and Losy, 2017). These disorders are, mainly, impairments in information processing speed (IPS) (Prakash et al., 2008; van Schependom et al., 2014), learning and episodic memory and executive functioning (Planche et al., 2016). In addition to cognitive impairments, recent studies have also identified social cognition impairments in PwMS (Cotter et al., 2016; Neuhaus et al., 2018). Social cognition encompasses the processes and knowledge involved in interpersonal interactions. Certain authors have argued that sociocognitive difficulties are likely to coexist with the abovementioned cognitive impairments in PwMS but may also arise independently (Golde et al., 2020). One area of social cognition that has been studied is moral judgment, which aims to understand the determinants of moral decision-making, including how and why individuals make moral choices in accordance with societal norms and expectations.

Moral judgment is commonly evaluated through a set of vignettes crafted by Greene and colleagues (Greene et al., 2001), which portray moral dilemmas. These dilemmas challenge participants to decide whether sacrificing one person is justifiable to save a larger group. Opting for such a sacrifice aligns with utilitarian reasoning, where the end justifies the means (e.g., sacrificing one life to save five is deemed acceptable). On the other hand, rejecting this option reflects deontological reasoning, asserting that sacrificing one to save many is morally wrong. In addition to the choice type (deontological or utilitarian), other commonly included measures in moral judgment assess the level of moral permissibility (with low scores reflecting *deontological* choice/reasoning), level of emotional reaction to the moral dilemma, and the degree of moral relativity, which gauges/estimates the extent to which others would act similarly (e.g., out of 100 people who responded to the dilemma, how many would respond as I did). In the MS population, research has shown that compared to control subjects, PwMS demonstrate a higher tendency for deontological moral choices in moral dilemmas (Ehrlé et al., 2020). They reported lower levels of moral permissiveness, along with an increase in both moral relativity and emotional reactivity (Gleichgerrcht et al., 2015; Realmuto et al., 2019).

Thus, it would appear that PwMS issue more deontological choices (decreased moral permissiveness) than controls. While PwMS seem to exhibit distinct patterns of moral reasoning, with a particular emphasis on deontological reasoning, the underlying factors driving these patterns/contributing to this specific pattern remain unclear. Considering that these patients otherwise exhibit empathy deficits as well as higher alexithymia than the global population (Chalah and Ayache, 2017; Ayache and Chalah, 2018), these patterns of results are surprising. Indeed, in other clinical populations, low empathic abilities and high alexithymia are linked to utilitarian rather than deontological moral judgments (Mendez et al., 2005).

## Aims

In this project, we aim to investigate motivational, cognitive and emotional factors that may explain deontological decision-making in PwMS.

One possible motivational factor that could explain these findings is the presence of a positivity bias in PwMS. A positivity bias is characterized by a heightened preference for positive emotions at the expense of negative emotions, resulting in a preference for processing positive information over negative information (Kauschke et al., 2019). This phenomenon has been extensively documented in the literature on cognitive aging in healthy individuals and has been shown to result in significant changes in emotional information processing with advancing age (Mather and Carstensen, 2003; Mill et al., 2009). In a meta-analysis of 100 studies (Reed et al., 2014), the authors report a consistent preference among older adults (compared to younger adults) for positive over negative information from various sources, such as faces (Mather and Carstensen, 2003; Leigland et al., 2004; Isaacowitz et al., 2006), labels (Piguet et al., 2008; Ready et al., 2017; Zhang et al., 2019), and emotional musical excerpts (Parks and Clancy, 2014).

Positivity bias may serve as an explanatory factor for the decision-making patterns observed in moral judgments in MS. According to motivational hypotheses presented in the literature, positivity bias is not underpinned by chronological age but rather by motivational changes linked to modifications of future temporal perspectives (Mather and Carstensen, 2003). If the emergence of a positivity effect is due to motivational changes, it is reasonable to think that various other factors and social contexts may also promote this effect. In fact, the positivity effect has been observed in chronic disease contexts, such as cancer (Dow et al., 1999; Petrie et al., 1999; Horgan et al., 2011; Schroevers et al., 2011) and HIV (Milam, 2004). Similarly, MS, which is the most common chronic disabling neurological condition among young adults, could lead to a shift in patients’ personal priorities, goals, and future projections. Supporting this hypothesis in MS, a study (Król et al., 2015) showed that patients had a different temporal orientation than controls, focusing more on the present. These findings suggest that a positivity bias may occur in MS patients and be responsible for some neuropsychological symptoms, particularly affecting emotional processing.

In this project, we aim to investigate whether a positivity bias could account for the more deontological decision-making patterns observed in MS (Gleichgerrcht et al., 2015; Realmuto et al., 2019; Ehrlé et al., 2020). Previous studies have shown that older individuals tend to make more ethical moral judgments than younger adults (McNair et al., 2019). Similar results have been observed in young individuals when their time horizons are experimentally restricted (Trémolière et al., 2012). Therefore, our research aims to examine whether the more deontological moral choices made by MS patients can be explained by changes in future temporal perspectives and a positivity bias.

On the cognitive front, the literature has shown that utilitarian judgments demand a higher level of cognitive resources and are thus less likely to occur when individuals are already under cognitive load (Greene et al., 2008; Moore et al., 2008). Consequently, another potential explanation for the predominantly deontological choices made by PwMS could be an optimization of limited cognitive resources. This hypothesis draws its roots from Greene’s dual-process theory (2005). According to Greene (2005), moral dilemmas can be categorized into two forms: “personal” dilemmas and “impersonal” dilemmas. Personal dilemmas involve the individual directly, presenting a scenario in which a moral rule is breached, leading to physical or emotional harm to others that can be directly attributed to the individual. On the other hand, “impersonal” dilemmas correspond to situations where the individual’s decision does not directly cause harm to others. Both types of dilemmas are illustrated below in examples 1 and 2.

Example 1 - personal dilemma:

A runaway trolley is speeding down the tracks towards five workmen who will be killed if the trolley continues on its present course. You are standing next to the tracks, but you are too far away to warn them. Next to you there is a very large stranger.

If you push the large stranger onto the tracks, the trolley will slide off the tracks and won’t continue its course towards the workmen. This will kill the stranger, but you will save the five workmen.

Do you cause the trolley to derail pushing the stranger onto the tracks, so the trolley does not reach the five workmen?

Example 2 – impersonal dilemma:

You are part of a shipyard dock team that attaches crane cables to containers to unload the cargo ships. You and the others have just attached cables to a container and are now climbing on top of it to make sure it is unloaded properly. Suddenly the red warning light flashes indicating that a cable is about to fail. If it fails over the deck the container will collapse onto five crewmembers.

If you push the emergency release button the container will be dropped back into the cargo bay. You and the others will be held suspended in mid air by your safety harnesses, but one crewmember is still working in the cargo bay. Dropping the container back there will kill him, but it will save the five crewmembers on the deck.

Do you drop the container pushing the emergency button, which will cause the container to fall back into the cargo bay on the crewmember, so the container won’t collapse onto the five crewmembers on the deck?

In personal moral dilemmas, “deontological” responses occur much more rapidly than “utilitarian” responses, indicating extremely swift emotional processing. This swift response time is not observed in “impersonal” moral dilemmas, where decision times are significantly longer. This supports the notion of a cognitive process involving the analysis of the costs and benefits of one’s actions (Greene et al., 2001, 2004).

Greene et al. (2008) also demonstrated that introducing an interfering task during the resolution of moral dilemmas could lead to an overload of participants’ moral decision-making processes, resulting in a decrease in available attentional resources. The decrease in attentional resources caused by overload interfered with the reasoning process and more specifically with utilitarian responses. Thus, there was an increase in the response times associated with these types of judgments. Notably, the findings from Greene’s study (2008) indicated that in the “dual task” condition, response times for “utilitarian” choices were longer than those associated with “deontological” choices, which do not necessitate cognitive resources. This study was conducted to provide empirical support for Greene’s dual-process theory of moral judgment.

Other researchers have also employed a similar approach to emphasize the influence of cognitive load on moral decision-making. For instance, authors manipulated the time constraints imposed on participants to respond to various moral dilemmas, creating an “under pressure” condition with a mere 8-s response time and a “without pressure” condition allowing 3 min for response (Suter and Hertwig, 2011). The results revealed that in the 8-s response time condition, most participants tended to provide deontological answers. Consequently, the authors concluded that when shorter decision times are enforced, individuals have limited time to engage in the cognitive processes inherent to their judgment, making them more inclined to make “ethical” choices. Based on these findings, it is plausible to hypothesize that cognitive impairments experienced by PwMS could lead to a preference for deontological choices. This preference may arise from the restricted cognitive resources available to them during moral dilemma situations, as demonstrated by the limitations in their cognitive processing abilities.

From an affective and emotional point of view, the prevalence of alexithymia in MS varies between studies, authors and tools used. Studies using the TAS-20 (Toronto Scale of Alexithymia) test estimate the prevalence of alexithymia at between 40 and 50% in the MS population (Chahraoui et al., 2013). Some authors suggest that alexithymia results from disturbances in interhemispheric transfer and could have an impact on social cognition (Degraeve et al., 2022). Previous studies have also shown that people suffering from MS have higher levels of emotional reactivity than healthy subjects when confronted with moral dilemmas (Gleichgerrcht et al., 2015; Patil et al., 2017; Realmuto et al., 2019) The literature has pointed out that deontological moral thinking is driven by strong emotional reactions: when emotions are experienced intensely, they are likely to override the decision-making process, resulting in a deontological moral judgment where causing harm is seen as morally wrong (Greene et al., 2001, 2004). fMRI investigations conducted by Greene et al. (2001, 2004) showed, for example, that “personal” dilemmas elicit stronger emotional reactions as well as more deontological judgments. However, all studies investigating emotional processing in MS have thus far focused exclusively on behavioral measures, with no studies combining these subjective measures with objective measures of the emotional response during a moral judgment task, such as electrodermal response (EDA) (Posada-Quintero and Chon, 2020). EDA refers to electrical variations in the skin associated with the functioning of the sweat glands, which are activated by nerve discharges of central origin under the control of the sympathetic nervous system. Numerous studies have demonstrated the usefulness of EDA in cognitive neuroscience, including studies of somatic marker theory and its relation to decision-making (Damasio, 1990), behavioral anticipation processes (Amiez et al., 2003), detection of reasoning bias (Carbonnell et al., 2006), and mental load (Salvia et al., 2012). Recent research has focused on clarifying the role of EDA in exploring emotional dimensions, as a reliable marker of reticular system functioning. Thus, the amplitude of EDA increases linearly with the estimated intensity of the emotional response and differentially with the valence of the emotional information (pleasant or unpleasant) (D’Hondt et al., 2013). Including an objective assessment of emotional reactivity in moral decision-making will provide a fine-grained and novel measure of patients’ emotional responses during choices and allow us to determine if PwMS have a higher level of emotional reactivity than controls. We will thus examine whether the higher tendency for deontological moral choices in moral dilemmas is linked to emotional experience in PwMS and more specifically, to a stronger emotional reactivity to moral dilemmas.

## Methods and analysis

### Objectives

The primary objective of this study is to compare the deontological or utilitarian choices made by patients and control individuals in moral dilemma situations using a set of 20 vignettes from Christensen et al. (2014) French-validated battery.

Three secondary objectives are associated with this protocol, corresponding to the three explanatory factors investigated (motivational, cognitive-affective, and emotional):

1. To assess whether deontological decision-making preference in PwMS (Ehrlé et al., 2020) can be attributed to a positivity bias.
2. To investigate the extent to which the presence of cognitive impairments contributes to patients’ decision making.
3. Evaluate the relationship between subjective and objective emotional reactivity and their impact on decision-making in PwMS.

### Participants

We conducted an *a priori* power analysis (Statistical and Analysis, 1992), to determine the appropriate sample size for our study. Since our primary endpoint relied on the nature of the choices made in the moral judgment task, specifically the measure of “moral permissiveness,” we used the data reported by Realmuto et al. (2019) to estimate the number of participants needed in each group. We aimed to achieve a minimum significance level of *p* = 0.05 and a power of 0.95. Based on the analysis of moral choices made in MS patients compared to a matched healthy population, we used the effect size reported in the Realmuto et al. (2019) study of *d* = 0.74 as an estimate. The analysis showed that we need to recruit at least 41 participants per group to have a 95% chance of detecting an effect. A safety margin of 10% has been added, so we plan to include 45 participants per group, which increased the chance of detecting the investigated effect to 96.7%.

However, it is important to note that this sample size is primarily designed to detect group differences and may not be sufficient for reliable moderated mediation analysis. Based on power analysis for moderated mediation with a medium effect size ( $f^{2}$ = 0.20), a power (1 – β) of 0.95 and a significance level of 0.05, and considering 3 predictors (i.e., main predictor, moderator, and their interaction), we estimate that approximately 90 participants in total are required. Since we plan to include 45 participants per group, totaling 90 participants, our sample size should be sufficient to ensure robust and reliable results for this analysis as well. However, we recommend including more participants during the data collection phase if feasible to further enhance the study’s robustness.

### Inclusion criteria

#### MS patient group

- Men or women aged 18–55 years.
- MS patient group: diagnosed with relapsing–remitting MS (RRMS) according to the 2010 McDonald criteria (Polman et al., 2011)
- EDSS score ≤ 4, with no significant motor, cerebellar or somesthesia disorders of the upper limbs, and no visual disorders (specific parameter of EDSS score less than 2) without relapses in the preceding 6 weeks. Fluent in French and able to express themselves clearly.
- Willing and able to understand and sign the informed consent and information letter regarding participation in the study.
- Health insurance coverage.

#### Control group

- Men or women aged 18–55 years.
- No known overall cognitive impairment and MoCA >26
- Fluent in French and able to express themselves clearly.
- Willing and able to understand and sign the informed consent and information letter regarding participation in the study.
- Health insurance coverage.

### Exclusion criteria

#### MS patient group

- Prior neurological pathology, head injury with loss of consciousness, or psychiatric pathology (apart from stable mild to moderate depressive symptoms)
- Severe general medical conditions that could affect participation in the study
- Perceptual or dysarthric disorders that could interfere with verbal communication or reading
- Severe cognitive impairment with an SDMT score of less than −2.5 standard deviations
- Severe depressive syndrome with a BDI-FS score > 10
- Visual and auditory impairments that could interfere with neuropsychological testing
- Major cerebellar syndrome or sensitive deficits
- Treatment with psychotropic drugs, except for benzodiazepines and hypnotics

#### Control group

- Prior neurological pathology, head injury with loss of consciousness, or psychiatric pathology (with the exception of stable mild to moderate depressive symptoms)
- Severe general medical conditions that could affect participation in the study
- Perceptual or dysarthric disorders that could interfere with verbal communication or reading
- Severe depressive syndrome with a BDI-FS score > 10
- Visual and auditory impairments that could interfere with neuropsychological testing
- Major cerebellar syndrome or sensitive deficits
- Treatment with psychotropic drugs, except for benzodiazepines and hypnotics

The study will be proposed to eligible participants at Saint Philibert or Saint Vincent de Paul Hospital in the following scenarios: during a routine neurology consultation, during a routine physical medicine and functional rehabilitation consultation or during inpatient hospitalization for disease-modifying therapy administration. Participants will be provided with an information letter explaining the study purpose and procedures, potential benefits and risks, study staff contact information and the consent form. Control subjects matched for sex, sociocultural level and age (+/− 2 years) will be recruited from the general population through a poster campaign. All data will be collected at either Saint Philibert hospital or Saint Vincent de Paul hospital, both situated in Lille, as requested by the ethic committee.

### Material

### Clinical and demographic assessment

The following clinical and demographic data will be collected: age, sex, years of education, laterality, and medical history by an interview preceding the experiment, MS subtype, disease duration, date of the last relapse and current disease-modifying treatment. Physical disability will be assessed using the *Expanded Disability Status Scale* (EDSS) (Kurtzke, 1983).

### Primary outcome measure

The moral judgement task includes 20 moral dilemma vignettes from Christensen et al.’s (2014) French-validated battery (Christensen et al., 2014), including 10 personal dilemmas and 10 impersonal dilemmas. For each vignette, participants will be asked to make a choice between deontological or utilitarian options, which will constitute our primary outcome measure. In addition to the choice type (deontological or utilitarian), other measures will be included:

- *Participants’ levels of moral permissiveness:* for each scenario presented, participants will be asked to rate on an 11-point Likert scale ranging from “*Not at all*” to “*Totally*” to what extent they find the proposed solution acceptable. An average score per scenario type (personal or impersonal) will be calculated.
- *Participants’ levels of moral relativity:* for each scenario presented, participants will be asked to rate, using an 11-point Likert scale ranging from “0” to “100”, the extent to which they consider the choice made to be consensual. An average score will be calculated for each type of scenario (personal or impersonal).
- *Participants’ subjective levels of emotional reactivity:* using an 11-point Likert scale, participants will be asked to rate their emotional response to each presented situation on a scale ranging from “*None*” to “*An intense emotion*.”

### Secondary outcomes measures

#### Cognitive and affective assessment

To investigate how cognitive and affective impairments contribute to patients’ decision-making, a routine cognitive and affective assessment will be administered to all patients. The administered tests as part of the cognitive and affective assessment are listed in Table 1, and a detailed description of each test is provided in Supplementary File S1.

**Table 1:** Cognitive and affective assessment.

**Cognitive assessment**	**Cognitive functions**	**Measures**
BICAMS		
CVLT	Verbal episodic memory	total free recall (/60)
BVMT-R	Visuo-spatial episodic memory	total free recall (/12)
SDMT	IPS	Number of correct substitutions
Digit span: Forward (DSF), Backward (DSB), Sequencing (DSS)	Working memory	Level of the span Total number of correct responses
Letter-Number Sequencing		
SCWT	Inhibition	Time to complete each condition Total number of errors
TMT	Reactive flexibility	Time to complete each condition Total number of errors
Verbal fluencies	Spontaneous flexibility	Number of different words.
PASAT	IPS/working memory	Number of correct responses (/60)

**Affective assessment**	**Affective dimensions**	
BDI-FS	Depression	Score ranging from 0 to 21
STAI-Y	Anxiety	Score ranging from 20 to 80
TAS-20	Alexithymia	Score ranging from 0 to 100
EQ8	Empathy	Score ranging from 0 to 80

BICAMS, Brief International Cognitive Assessment for Multiple Sclerosis; CVLT, California Verbal Learning Test; BVMT-R, Brief Visuo-spatial Memory Test; SDMT, Symbol Digit Modalities Test; SWCT, Stroop Color-Word Test; TMT, Trail Making Test; PASAT, Paced Auditory Serial Addition Test; BDI-FS, Fast-Screen Beck Depression Inventory; STAI-Y, State Trait Anxiety Inventory.

#### Motivational assessment

The assessment of participants’ explicit temporal perspective will be conducted using the French adaptation of the *Future Time Perspective Scale*, developed by Carstensen and Lang (1996) and adapted by Sanfourche-Gaume et al. (2022). Participants will rate their level of agreement on 10 items using a 5-point scale ranging from 1 (*not at all*) to 5 (*completely*) to assess their future time perspective (e.g., “I plan to set many new goals in the future”). A total score will be calculated. To assess participants’ implicit temporal perspective, a word completion task (Trémolière et al., 2012) will be used. The task presents participants with 20 gap words that can be completed with either words referring to restricted future time perspective (“target words”) or neutral words. For example, the word “COFF _ _” can be completed with “COFFIN” or “COFFEE.” The percentage of target words completed will be calculated. We anticipate no disparity between implicit and explicit assessment of temporal perspective. However, employing a dual modality could help us mitigate the potential influence of a social desirability bias. Consequently, we plan to conduct both joint and separate analyses. If implicit scores are higher, it is possible that social desirability and/or a lack of insight could affect how patients evaluate their temporal perspective.

To assess attentional preferences for positive information, also known as positivity bias, a French adaptation of the “Dot Probe Task” (MacLeod et al., 1986) will be used. This detection task measures attentional preferences for positive or negative information, allowing the evaluation of whether individuals tend to focus more on positive or negative information. In the Dot Probe Task, participants are asked to identify the laterality of a dot-shaped target as quickly as possible. Prior to the target’s appearance, a static image of a face showing a negative or positive emotion appears on each side of the screen for a limited time. Trials where the face showing a positive emotion appears on the same side as the target are considered congruent trials. Conversely, when the face presenting a positive emotion appears on the opposite side to the target, the trial is considered incongruent. The positivity bias is calculated using the difference, if any, between the average reaction times for congruent and incongruent trials: Positivity Bias = RT incongruent (joy) − RT congruent (joy).

To better isolate the positivity bias and ensure that the observed bias is not influenced by a general emotional response but is specifically related to an attraction to positive stimuli, analyses will also be conducted using an alternative calculation method for positivity bias, considering the differences in reaction times for both positive and negative faces: Positivity Bias = [Pos(congruentRT - incongruentRT)] -[Neg(congruentRT - incongruentRT)].

#### Physiological data

During the moral judgment task, electrodermal responses will be recorded through the Biosignalsplux Researcher® equipment, which is certified for medical research and has been used in previous studies (Muñoz et al., 2017, 2018; Crowell et al., 2020). The signal will be recorded at a sampling frequency of 200 Hz using Biosignalsplux Researcher® equipment connected to a computer hosting *Biosignalplux* software. The EDA will be collected with two pregelled self-adhesive disposable Ag/AgCl electrodes, filled with an isotonic conductive paste (0.05 M NaCl) and placed at the level of the second phalanx of the index and middle fingers of the nondominant hand (D’Hondt et al., 2013).

The purpose of using this device is to measure the participant’s objective emotional reactivity during the task. The signals recorded by the device will be examined relative to the participant’s own baseline levels (measured without stimulus, i.e., baseline measure), which will serve as a reference for interpreting the signals obtained during the task.

## Procedure

The experiment will be conducted in a single session, which will last approximately 2.5 h and will include, in the following order, the administration of a consent form and a demographic questionnaire; cognitive tests and affective questionnaires (a full description of the material used is available in supplementary material 1), tasks to assess motivational factors (FTPS, Dot probe task, word completion task) and a moral judgment task coupled with a measurement of EDA. Twenty moral dilemma vignettes from the work of Greene et al. (2004) will be presented to the participants. For each scenario, participants must make either a utilitarian choice or a deontological one. During the moral judgment task, an electrodermal response recording device will be placed on the participant’s nondominant hand using two electrodes to objectively measure emotional reactivity. Prior to starting the moral judgment task, a baseline measurement of the electrodermal response will be taken for 1 min while the participant is instructed to relax.

### Participant timeline

The detailed course of the study for every participant is presented in Table 2.

**Table 2:** Participant timeline.

	**Enrolment**	**Allocation**	**Post-Allocation**
TIMEPOINT	***7 days before allocation***	**0**	***60 days maximum after***
ENROLMENT:			
Eligibility screen	X		
Informed consent	X		
Allocation		X	
ASSESSMENTS:			
Clinical and demographic assessment			X
*Cognitive assessment*			X
*Psychological assessment*			X
*Moral judgement assessment*			X
*Time perception assessment*			X
*Physiological assessment*			X

## Statistical analysis

### Descriptive

First, we will conduct a description of each group of participants (i.e., MS patients; healthy controls) through the responses provided to the demographic questionnaire, and the cognitive and affective evaluation. For qualitative variables, the size and frequency of each category will be calculated (e.g., number of men and women, number of participants by level of education). For quantitative variables, the mean and standard deviation will be calculated (e.g., age of participants, laterality, and disease duration for PwMS). Additionally, independent samples *t-*tests —or nonparametric Mann- Whitney *U* tests when appropriate— will be conducted to analyze group differences in quantitative demographic measures. Group differences on demographic qualitative measures will be analyzed using chi-squared tests.

### Group differences in main outcome measures (differences in moral choices)

To compare moral choices made by patients vs. controls in moral dilemmas, we will examine the differences in moral choices (deontological vs. utilitarian) between groups using a mixed model approach. The independent variables will include the group (PwMS vs. controls) as a between-subjects factor and the type of scenario (personal vs. impersonal) as a within-subjects factor. The dependent variable will be the percentage of deontological choices. When appropriate, follow-up pairwise comparisons will be conducted with Bonferroni multiple comparisons correction.

### Group differences in the second outcome measures (three explanatory factors of the observed differences in moral choices)

#### Motivational hypothesis: *t*-tests, regressions and moderated-mediation analysis

In order to compare time perspective and attention preferences for positive information between groups, we will conduct independent samples t-tests—or nonparametric Mann- Whitney *U* tests when appropriate. In the MS group, we will additionally explore whether these scores are correlated with age, disease duration, EDSS, date of the last relapse, cognitive status (using BICAMS scores) and affective status (using BDI-FS scores).

We will next examine whether time perspective scores (both explicit and implicit, see “Temporal perception assessment” section) predict attentional preferences for positive information and deontological moral choices (specifically, percentage of deontological choices) for PwMS and controls. This will be done using two separate multiple regression models for each dependent variable, with time perspective scores and group as the predictor variables and attentional preferences for positive information and deontological moral choices as the respective dependent variables.”

Finally, to investigate whether the positivity effect mediates the association between patients’ deontological preferences and restricted temporal perspectives, we will test the existence of a conditional indirect effect (a moderated-mediation effect). Based on existing literature, we expected attentional preferences for positive information to mediate the relationship between temporal perspective and deontological moral choices (specifically, percentage of deontological choices). As we expect an interaction effect between temporal perspective and participant group (PwMS vs. controls), we expect to observe a mediated moderation effect (Muller et al., 2005). This means that the influence of the temporal perspective on deontological moral choices should be moderated by the participant group, and this moderation should be mediated by attentional preferences for positive information. Note that this relationship is expected to be observed only for “impersonal” scenarios. Although moderated mediation can suggest complex relationships among variables, it cannot confirm causality. Longitudinal studies would be more appropriate to explore these causal relationships further.

#### Cognitive-affective hypothesis: Regression analyses

To assess the extent to which cognitive and affective impairments contributes to patients’ decision-making, we will conduct multiple regression analyses. These analyses will aim to determine whether cognitive and affective scores (more specifically alexithymia and empathy scores), as detailed in Table 1, predict the proportion of deontological choices in both personal and impersonal moral dilemmas and will help to investigate the relationships between decision-making patterns and the level of cognitive and affective disorders in patients.

#### Neurophysiological hypothesis: correlation analyses

To investigate the extent to which the subjective emotional reactivity recorded during the moral judgment task is associated with objective responses, we will calculate correlations between the subjective and objective levels of emotional reactivity during the moral judgment task (i.e., reported levels versus individuals’ EDA) for each type of scenario (personal, impersonal) for patients and controls separately. We will next test the significance of the difference between these two correlations using Fisher’s *z*-Test. This analysis will help to investigate whether patients’ deontological preferences are associated with increased emotional reactivity.

### Data monitoring and management

The trial will be monitored by a Data Monitoring Committee. Investigators will complete an electronic Case Report Form (e-CRF). Access to data will be limited to the two co-investigators of the study. Data can only be modified by a study investigator or a collaborator designated by the investigator. Data will be managed in compliance with Data Protection Act of January 6, 1978 (*Loi Informatique et Libertés, Commission Nationale Informatique et Liberté;* CNIL). To protect the participants’ privacy, a unique patient identification number will automatically be assigned to each participant. Only de-identified data will be recorded in the database. All data are entered and stored into a centralized secure electronic data management system (OpenClinica) created in accordance with good clinical practice. Access is only possible using an loggin and secure password. The password must contain a minimum of 8 characters, including at least one lower case letter, one upper case letter, one number and one symbol. The session will be automatically disconnected after 15 min of inactivity. All connections and attempts to connect are logged. Once the final analysis has been completed and validated, all data will be archived for at least 15 years, in accordance with the Public Health Code Decree of 11 August 2008.

## Discussion and expected results

Based on our study design and hypotheses, we expect to observe a higher frequency of deontological responses to moral dilemmas in PwMS compared to control participants, who are likely to exhibit more utilitarian responses. Additionally, we anticipate the level of cognitive impairment to predict the frequency of deontological responses to the moral dilemma task. This may indicate that patients with cognitive decline rely more heavily on automated decision-making mechanisms. At the same time, we expect the level of emotional arousal to predict the frequency of deontological responses. This finding could indicate that people with higher emotional reactivity tend to rely more on an emotional process than on a cognitive process when faced with a moral dilemma.

Finally, we anticipate a stronger positivity bias in PwMS, indicative of a temporal perspective bias. This bias is expected to correlate with an increased frequency of deontological responses in the moral dilemma task, with participants demonstrating a significant positivity bias making deontological choices more often. Consequently, we anticipate the emergence of a significant moderated-mediation effect within the MS group, between temporal perspective, attentional preferences, and moral positioning.

The results of this study examining the impact of positivity bias, cognitive impairment, and alteration of emotional processes on deontological moral reasoning in PwMS could have important implications for comprehending their decision-making, social interactions, and overall well-being. Deontological moral reasoning, which prioritizes the observance of one’s moral principles rather than the common good, can have an impact on different facets of an individual’s decision-making and everyday conduct. By clarifying the elements that contribute to this form of ethical reasoning in PwMS, we can get significant understanding into their decision-making tendencies. This comprehension could assist healthcare professionals, carers, and relatives in providing enhanced support and adapting to the distinct requirements and viewpoints of individuals with MS. This study aims to elucidate the difficulty faced by PwMS in navigating social circumstances by identifying the factors that influence their distinctive decision-making process when confronted with decisions of significant moral importance.

The results of this study could provide valuable insights for creating specific therapies and support techniques that aim to improve social cognition and facilitate more effective decision-making in PwMS, enhancing the overall well-being and quality of life for these individuals.

## Ethics statement

The studies involving humans were approved by Comité de Protection des Personnes Ouest III National number 2023-A00447-38. The studies were conducted in accordance with the local legislation and institutional requirements. The participants provided their written informed consent to participate in this study.

## Author contributions

LZ: Visualization, Project administration, Funding acquisition, Writing – review & editing, Writing – original draft, Methodology, Investigation, Data curation, Conceptualization. BD: Writing – review & editing, Writing – original draft, Visualization, Validation, Supervision, Project administration, Methodology, Investigation, Funding acquisition, Data curation, Conceptualization. AP: Writing – review & editing, Visualization, Validation, Supervision, Project administration, Methodology, Conceptualization. JP: Writing – review & editing, Resources, Investigation, Data curation. LN: Writing – review & editing, Methodology. AK: Writing – review & editing, Resources, Investigation, Data curation. CD: Writing – review & editing, Resources, Investigation, Data curation. BL: Writing – review & editing, Visualization, Validation, Supervision, Project administration, Methodology, Investigation, Funding acquisition, Formal analysis, Data curation, Conceptualization.
