# Supplementary material for: Distinguishing the role of positivity bias, cognitive impairment and emotional reactivity in the deontological preference in multiple sclerosis during moral dilemmas: a social cognition study protocol
Source: Front Psychol. 2024 Jul 18;15:1404876. doi: 10.3389/fpsyg.2024.1404876 (PMC11291456; doi:10.3389/fpsyg.2024.1404876)
Supplement: Supplementary file 1 [file Data_Sheet_1.PDF]

Supplementary data 1: Measures of moral judgment, neuropsychological and psychological assessment

Secondary outcomes measures

Cognitive assessment

The Brief International Cognitive Assessment for Multiple Sclerosis (BICAMS) [2] includes the following 3 cognitive measures:

► The *California Verbal Learning Test* (CVLT) [3], a verbal memory and learning test which consists of 16 words presented in a fixed order to the participant over 5 consecutive trials. The 16 words contain 4 words in each of the 4 distinct categories. On each trial, participants are required to recall as many words as possible after each presentation of the list. The dependent variable is the total number of correct responses across the 5 trials.

► The *Brief Visuo-spatial Memory Test* (BVMT) [4], a visual memory and learning test which requires participants to encode six geometrical figures and memorize their precise location during a 10s presentation. Immediately afterwards, participants have to draw the memorized figures in the right location. The procedure is repeated three times. Depending on figure and exact location accuracy, a scoring from zero to two points for each figure is given. The total recall score consists of the sum of the participants' scores of the three trials.

► The *Symbol Digit Modalities Test* (SDMT) [5], which provides an index of information processing speed, requires participants to orally substitute digits for geometric symbols shown in a key sequence of nine symbols as quickly as possible. The dependent variable is the number of digits correctly substituted for symbols in 90 seconds.

► *Direct and forward digit spans* will assess, respectively, short-term memory and working memory. Participants heard a list of digits with instructions to repeat the digits immediately in the same order (direct span) or in the reverse order (forward). The task has six progressive levels of difficulty that start with two digits and build up to seven, with a pair of digit strings at each level. Participants had to repeat one string at each level correctly to proceed to the next level. Testing stopped when both strings at one level were incorrect. In each condition, the level of the span and the total number of correct responses will be measured.

►The *Letter-Number Sequencing* is a core working memory task and measures attention span, short-term auditory recall, processing speed and sequencing abilities. The task involves listening to and remembering a string of digits and letters read aloud at a speed of one per second, then recalling the information by repeating the numbers in chronological order, followed by the letters in alphabetical order.

► *Stroop Color-Word Test* [6,7] to assess the inhibition processing. Subjects are required to read three different tables as fast as possible. 1) in the two firs congruous condition, participants are required to name different colour patches and to read names of colours printed in black ink, 3) the third table, incongruent condition, participants are required to name the colour of the ink instead of reading the word. The performance is evaluated by the time to complete each part and the total number of errors.

►*Trail Making Test* [8,9], to assess reactive flexibility, through two conditions: Part A, the subject have to link numbers between 1 to 25, in ascending order, as quickly and as precisely as possible; Part B, the subject have to connect the items by alternating the numbers (1to 13) and the letters (A to L) in ascending order of the numbers and in the normal order of the alphabet (1A, 2B, 3C, ...). The performance is evaluated by the time to complete each part and the total number of errors.

► *Categorical and phonemic verbal fluency* is used to assess spontaneous flexibility abilities. For 2 minutes, the subject is asked to produce the most words belonging, firstly, to a certain semantic category (Animals), then secondarily, starting with the same letter (P). Performance is assessed by the number of different words.

►*The Paced Auditory Serial Addition Test* [10] is used to assess IPS and working memory. A serie of 61 singles digits from 1 to 9 were randomly delivered at presentation rates of one number every three seconds. Subject have to add each digit to the one immediately preceding it: the second had to be added to the first, the third to the second, and so on. The analysed variables of the PASAT were the number (and the percentage) of correct additions (maximum score 60) and dyad scores (two consecutive correct answers, maximum score 59).

Psychological assessment

► *TAS-20 alexithymia scale* [11]*:* this scale will be administered to assess the level of alexithymia in participants. This self-reported questionnaire consists of 20 items and evaluates three factors: difficulty in identifying and describing feelings, externally oriented thinking, and reduced imaginative processes.

► *EQ-8 Empathy* Scale [12]: will be used to measure the level of empathy in participants. This self-report questionnaire consists of 8 items and participants are asked to rate their agreement with each item on a scale of 1 to 5 (1 = *completely disagree*, 5 = *completely agree*).

►*Fast-Screen Beck Depression Inventory (BDI-FS)* [13] is a screening tool for depressive symptoms, which avoid the considerable overlap between the vegetative symptoms of depressive disorder and the neurological manifestations of MS (e.g., insomnia, fatigue, weight loss, poor concentration), containing 13 items.

► *State Trait Anxiety Inventory (STAI-Y)* [14]: this self-report questionnaire measures the presence and severity of current symptoms of anxiety and a generalised propensity to be anxious. There are two subscales: 20 items allocated to each of the State Anxiety (S-Anxiety) and Trait Anxiety (T-Anxiety).

References

[1] Benedict RH, Amato MP, Boringa J, Brochet B, Foley F, Fredrikson S, et al. Brief International Cognitive Assessment for MS (BICAMS): international standards for validation. BMC Neurol 2012;12:55.

[2] Delis D, Kramer J, Kaplan E, Ober B. California Verbal Learning Test. TX : The Psychological Corporation. San Antonio: 1987.

[3] Benedict HB. Brief visual memory test-revised: Professional manual. Odessa FL Psychol Assess Resour 1997.

[4] Smith A. Symbol digit modalities test. Western psychological services Los Angeles; 1973.

[5] Stroop JR. Studies of interference in serial verbal reactions. J Exp Psychol Gen 1992;121:15–23. https://doi.org/10.1037/0096-3445.121.1.15.

[6] Fonctions exécutives et pathologies neurologiques et psychiatriques. Evaluation en pratique clinique - GREFEX,Olivier Godefroy. n.d.

[7] Tombaugh T. Trail Making Test A and B: Normative data stratified by age and education. Arch Clin Neuropsychol 2004;19:203–14. https://doi.org/10.1016/S0887- 6177(03)00039-8.

[8] Shallice T. Specific impairments of planning. Philos Trans R Soc Lond B Biol Sci 1982;298:199–209. https://doi.org/10.1098/rstb.1982.0082.

[9] Shallice T, Burgess PW. Deficits in strategy application following frontal lobe damage in man. Brain J Neurol 1991;114 (Pt 2):727–41. https://doi.org/10.1093/brain/114.2.727.

[10] Tombaugh TN. A comprehensive review of the Paced Auditory Serial Addition Test (PASAT). Arch Clin Neuropsychol Off J Natl Acad Neuropsychol 2006;21:53–76. https://doi.org/10.1016/j.acn.2005.07.006.

[11] Bagby RM, Taylor GJ, Parker JD, Dickens SE. The development of the Toronto Structured Interview for Alexithymia: item selection, factor structure, reliability and concurrent validity. Psychotherapy and psychosomatics. 2005 Dec 16;75(1):25-39.

[12] Loewen PJ, Lyle G, Nachshen JS. An eight-item form of the Empathy Quotient (EQ) and an application to charitable giving. Retrieved from crcee. umontreal. ca/pdf/Eight% 20Question% 20ES_final. pdf. 2009.

[13] Benedict RH, Fishman I, McClellan MM, Bakshi R, Weinstock-Guttman B. Validity of the Beck Depression Inventory-Fast Screen in multiple sclerosis. Mult Scler J 2003;9:393– 6. https://doi.org/10.1191/1352458503ms902oa.

[14] Spielberger C, Gorsuch R, Lushene R, Vagg P, Jacobs G. Manual for the State-Trait Anxiety Inventory (Form Y1 – Y2). vol. IV. 1983.
